# A Clinical Audit of Thyroid Hormonal Replacement After Total Thyroidectomy

**DOI:** 10.7759/cureus.50374

**Published:** 2023-12-12

**Authors:** Islam Mansy, Abdelfatah M Elsenosy, Eslam M Hassan, Mujtaba Zakria

**Affiliations:** 1 General Surgery and Surgical Oncology, Maadi Armed Forces Medical Complex, Cairo, EGY; 2 Trauma and Orthopaedics, University Hospitals Dorset, Poole, GBR; 3 Trauma and Orthopaedics, Poole General Hospital, Poole, GBR; 4 General Surgery, Cairo University, Cairo, EGY

**Keywords:** euthyroidism, hypothyroidism, tsh, total thyroidectomy, thyroid hormonal replacement

## Abstract

Background

Thyroid hormone replacement (THR) in athyreotic patients post-thyroidectomy due to thyroid cancer might seem like a straightforward clinical issue to address. To investigate the impact of THR on enhancing thyroid-stimulating hormone (TSH) levels, we conducted a clinical audit, tailoring the dosage based on patient weight and aligning with the standards outlined by the National Institute for Health and Care Excellence and the American Thyroid Association guidelines.

Methodology

This retrospective and prospective audit analyzed outpatient clinic records for hormone replacement therapy (HRT) post-total thyroidectomy. Retrospective data from March to May 2022 were collected, followed by prospective data after interventions adjusting HRT based on patient weight to digitize clinic notes. The second phase involved changes for 20 scheduled thyroidectomy patients among the total 37 included in the study.

Results

The thyroid profiles of both groups in the initial and subsequent cycles, treated with adjusted doses of THR, exhibited normal levels of thyroid hormones and calcium. No substantial differences were observed between the groups. On multivariate logistic regression analysis, we found that older age, male sex, body mass index, and preoperative TSH level were the only significant predictors of the need for hormonal therapy.

Conclusions

Optimal dose of THR after total thyroidectomy had a positive effect on TSH levels in hypothyroidism patients. Hence, THR should be prescribed according to patient weight based on standards and guidelines.

## Introduction

Over the past few decades, the incidence of thyroid cancer has significantly increased globally [1]. The incidence of thyroid cancer has risen dramatically in the United States over the past four decades, with similar patterns observed internationally. Thyroid cancer currently ranks as the 13th most common cancer diagnosis overall and the sixth most common among women. The most recent estimates provided by the American Cancer Society predict around 43,720 new instances of thyroid cancer in the United States in 2023, with approximately 12,540 occurring in men and 31,180 in women. Additionally, an estimated 2,120 fatalities due to thyroid cancer are expected, with roughly 970 in men and 1,150 in women [2].

The primary cause for this rise has been identified as papillary thyroid carcinoma, which accounts for up to 88% of all thyroid cancer cases and is the most prevalent histologic subtype of the disease [3]. The primary treatment for thyroid cancer is surgery. A total thyroidectomy or lobectomy can be used to describe the extent of the procedure. Benefits of total thyroidectomy include the ability to precisely monitor thyroglobulin levels after surgery, eliminate tiny cancer foci in the contralateral lobe, and employ radioactive iodine as an adjuvant therapy. Therefore, hormone replacement therapy (HRT) is essential to preserving the body’s basic processes [4].

Thyroid hormone replacement (THR) in athyreotic patients after thyroidectomy for thyroid cancer ought to be a reasonably simple clinical issue to resolve. As the thyroid has been removed and hormone levels are depleted, the doctor recommends a levothyroxine prescription to supplement the thyroid’s natural hormone production, resolving the problem [5].

Levothyroxine (L-T4) therapy is used to prevent thyroid hypofunction following surgery and to replenish insufficient thyroid hormones. Before thyroid-stimulating hormone (TSH) assays were developed, people with primary hypothyroidism were advised to take 200-400 µg of L-T4 daily [6].

The need to reevaluate postoperative replacement medication arises from the growing trend of performing total or near-total thyroidectomy for benign thyroid disease, as well as the sensitivity of TSH testing. Subclinical hypo and hyperthyroidism are examples of newly described entities; nevertheless, their pathogenic importance in the immediate postoperative period is yet to be determined [7].

While some authors support maintaining the current 4 mIU/L limit, others argue for reducing the normal range for serum TSH [8]. The benefits of mixing liothyronine with L-T4 for replacement therapy are being studied by other researchers [9].

A therapeutic strategy for patients with minimal residual thyroid function is recommended by the American Thyroid Association (ATA) and the American Association of Clinical Endocrinologists in their most recent guidelines for hypothyroidism. Replacement therapy of approximately 1.6 μg/kg/day of levothyroxine is advised. Patients with athyreogenesis (after total thyroidectomy and/or radioiodine therapy) and central hypothyroidism may require greater doses, as acknowledged by these recommendations [10]. The empirical dose for thyroid replacement medication, per customary practice, was 100-150 µg daily. Upon initial follow-up, euthyroidism was achieved by 21% to 37% of patients who adhered to this prescribed dosage regimen [11].

A University of Wisconsin meta-analysis compared standard and novel dosing schemes. The standard weight-based scheme predicted 51.3% of doses accurately, while the more novel Poisson regression scheme achieved a significantly higher accuracy at 64.8%. The Poisson regression scheme outperformed the best existing scheme based on body mass index (BMI)/weight, which predicted doses accurately at 60.9% compared to Poisson’s 64.8% (p = 0.031) [12].

Therefore, we implemented this clinical audit with a new dose according to patient weight relying on standards and guidelines outlined by the National Institute for Health and Care Excellence (NICE) and ATA, and aimed to assess the THR effect on the improvement of TSH levels.

## Materials and methods

In this retrospective and prospective closed-loop audit, the outpatient clinic notes were investigated for the HRT dose after total thyroidectomy. Audits were finished in three different cycles. The patients provided informed written consent before participating in the study. This study was conducted at the General Surgery and Surgical Oncology Department at Maadi Armed Forces Medical Complex. The study was done after obtaining approval from the Research Ethics Committee, General Surgery and Surgical Oncology Department, Maadi Armed Forces Medical Complex (approval number: AFMIC1904513).

In the initial phase, a retrospective collection of data encompassed all outpatient clinic notes from March 2022 to May 2022. Subsequently, a shift toward prospective data collection occurred, involving interventions such as adjusting HRT based on patient weight. This change aimed to facilitate the digitization of clinic notes. The dataset comprised thyroid profile results and symptoms associated with hypothyroidism from a cohort of 20 patients. The second phase commenced by implementing modifications on these 20 patients scheduled for total thyroidectomy. Changes were instituted, including an adjustment of levothyroxine dosage to 1.6 µg/kg, replacing the standard 100 µg. Additionally, the alterations encompassed regular thyroid profile assessments within a six to eight-week interval and inquiries about hypothyroidism symptoms during follow-up visits. These changes were implemented between May 2022 and July 2022, marking the completion of the second cycle [13].

Patients who were receiving levothyroxine for primary hypothyroidism, patients on levothyroxine after hemithyroidectomy, those on thyroid hormone supplementation before surgery, those 75 years of age, and patients with a lack of preoperative TSH availability were excluded from this study.

Then, data were collected from the re-audit from July to September 2022 retrospectively. Standards to implement were based on the recommendations of NICE and ATA guidelines. Data were collected from patients after total thyroidectomy.

Data were obtained on the following patient characteristics: age, sex, residence, weight, BMI, a history of thyroid disease in the family, preoperative TSH, euthyroid levothyroxine dosage, and postoperative symptoms (<3 months) and complications (<3 months).

According to our institution’s laboratory standards, the normal range for TSH was determined to be 0.464 to 4.679 mIU/L [13].

The problem was hypothyroidism symptoms and abnormal thyroid profile in patients after total thyroidectomy. Almost all patients needed dose adjustment.

According to NICE guidelines and ATA guidelines, HRT after total thyroidectomy was done according to patient weight.

Data collection and analysis involved initially gathering information retrospectively from a group of 20 patients. Among these, 17 patients exhibited abnormal results. Subsequently, these 17 patients were combined with an additional 20 new patients, resulting in a total cohort of 37 patients for the second cycle.

Implementation

Levothyroxine, prescribed at a rate of 1.6 µg/kg based on patient age, was initiated for all individuals within the third to fifth days following surgery, adhering to a specified protocol. The initial dosage was individualized considering their preoperative thyroid function and the extent of the surgical procedure. In our study, we tailored the initial HRT dosage by evaluating preoperative thyroid function, including TSH levels and hormone assays. We also customized dosages based on the type of surgery, i.e., total or partial thyroidectomy, using established protocols. Our approach aligned with the NICE and ATA guidelines, offering specific dosage recommendations for individualized post-thyroidectomy HRT initiation. No modifications to the dosage were made during the initial visit within 10-14 days post-surgery.

Blood samples were collected for analysis of TSH and free T4 index (FT4I). Adjustments to the levothyroxine dosage were performed as required, with incremental changes of 25-50 µg through dose titration. TSH measurements utilized a third-generation chemiluminometer (Immuno 1, Bayer, Germany) with a detection limit of 0.01 mIU/L and a functional sensitivity of 0.014 mIU/L. The manufacturer-defined normal range for TSH was 0.15-4.60 mIU/L.

Statistical analysis

SPSS version 28 (IBM Corp., Armonk, NY, USA) was utilized for statistical analysis. The unpaired Student’s t-test was utilized to compare the two groups based on quantitative data that were reported as mean and standard deviation (SD). The frequency and percentage (%) of the qualitative variables were reported, and when suitable, Fisher’s exact test or chi-square test was utilized for analysis. A statistically significant result was defined as a two-tailed p-value of less than 0.05. The link between a dependent variable and several independent factors was estimated utilizing multivariate logistic regression.

## Results

In the first cycle, 17 patients underwent thyroidectomy and received an unadjusted dose of HRT. During follow-up, we found that those patients developed abnormal thyroid profiles (low TSH levels) and were given an adjusted dose of HRT. In the second cycle, we included 20 patients who had thyroid cancer (with normal thyroid profile). These patients underwent thyroidectomy and received an adjusted dose of HRT after intervention according to weight. All patients (n = 37) were on the adjusted dose of HRT and had normal thyroid profiles (euthyroidism).

Regarding the baseline characteristics for the 17 patients who underwent thyroidectomy and had THR, there were five (29.4%) males and 12 (70.6%) females, with a mean age of 34.4 ± 10.32 years. The mean weight of the studied patients was 83.7 ± 7.36 kg, the mean height was 1.7 ± 0.06 m, and the mean BMI was 30.3 ± 3.74 kg/m^2^. Among the studied patients, 10 (58.8%) were from urban areas, and seven (41.2%) were from rural areas. There were six (35.29%) patients with a positive family history of thyroid diseases. The mean duration of the disease was 4.2 ± 1.2 years.

Regarding the TSH level, the mean preoperative TSH level was 1.9 ± 0.7 mIU/L. The mean postoperative TSH level after THR (unadjusted dose) was 0.29 ± 0.12 mIU/L. We observed that those patients had abnormally low levels of TSH after as they were on the unadjusted dose of THR (Table 1).

**Table 1:** Baseline characteristics of patients with abnormal thyroid function in the first cycle (n = 17). Data presented as mean ± SD or frequency (%). BMI: body mass index; THR: thyroid hormone replacement; TSH: thyroid-stimulating hormone

		Total (n = 17)
Age (years)		34.4 ± 10.32
Sex	Male	5 (29.4%)
	Female	12 (70.6%)
Weight (kg)		83.7 ± 7.36
Height (m)		1.7 ± 0.06
BMI (kg/m^2^)		30.3 ± 3.74
Residence	Urban	10 (58.8%)
	Rural	7 (41.2%)
Family history of thyroid diseases		6 (35.29%)
Duration of disease (years)		4.2 ± 1.2
Preoperative TSH level (mIU/L)		1.9 ± 0.7
TSH level after THR (unadjusted dose)		0.29 ± 0.12

The clinical examination of the vital signs revealed that the mean systolic blood pressure was 117.03 ± 11.99 mmHg, the mean diastolic blood pressure was 73.5 ± 9.49 mmHg, and the mean heart rate was 86.8 ± 8.99 beats/minute (Table 2).

**Table 2:** Clinical examination of the vital signs of patients with abnormal thyroid function in the first cycle (n = 17). Data presented as mean ± SD. SBP: systolic blood pressure; DBP: diastolic blood pressure; HR: heart rate

	Total (n = 17)
SBP (mmHg)	117.03 ± 11.99
DBP (mmHg)	73.5 ± 9.49
HR (beats/minute)	86.8 ± 8.99

Table 3 shows the demographic data of 20 patients involved in the second cycle with thyroid cancer who underwent thyroidectomy and received an adjusted dose of HRT after intervention according to their weight. There were five (25%) males and 15 (75%) females, with a mean age of 34.4 ± 10.3 years. The mean BMI was 24.3 ± 0.81 kg/m^2^. The mean duration of the disease was 3.7 ± 1.11 years. They had a normal preoperative TSH with a mean of 1.25 ± 0.11 mIU/L. After thyroidectomy and treatment with an adjusted dose of THR, they had a normal post-treatment TSH with a mean of 1.22 ± 0.12 mIU/L (Table 3).

**Table 3:** Baseline characteristics of the studied patients in the second cycle (n = 20). Data presented as mean ± SD or frequency (%). BMI: body mass index; THR: thyroid hormone replacement; TSH: thyroid-stimulating hormone

		Post-treatment second cycle (n = 20)
Age (years)		34.4 ± 10.3
Sex	Male	5 (25%)
	Female	15 (75%)
BMI (kg/m^2^)		24.3 ± 0.81
Duration of disease (years)		3.7 ± 1.11
Preoperative TSH (mIU/L)		1.25 ± 0.11
Post-treatment TSH level (mIU/L) after thyroidectomy and treatment with an adjusted dose of THR		1.22 ± 0.12

Table 4 shows the thyroid profiles and calcium levels after treatment of both patient groups with an adjusted dose of THR, revealing normal thyroid profiles and calcium levels with no significant difference between the groups.

**Table 4:** Post-treatment thyroid profiles and calcium levels of the studied patients. Data presented as mean ± SD; *: statistically significant at p-values <0.05. TSH: thyroid stimulating hormone; T4: thyroxin; T3: triiodothyronine

	Total (n = 37)	Patients with abnormal thyroid function in the first cycle (n = 17)	Patients in the second cycle (n = 20)	P-value
TSH (mIU/L)	1.19 ± 07	1.21 ± 0.08	1.22 ± 0.12	0.772
Free T4 level (mIU/L)	1.11 ± 0.07	1.10 ± 0.08	1.11 ± 0.06	0.667
Free T3 level (mIU/L)	1.62 ± 0.22	1.65 ± 0.21	1.59 ± 0.23	0.416
Calcium level (mg/dL)	7.74 ± 0.20	7.70 ± 0.22	7.77 ± 0.19	0.306

Regarding the postoperative complications, temporary vocal cord weakness was reported in four (10.8%) patients, and hematoma was reported in two (5.4%), whereas permanent vocal cord weakness and seroma did not occur in any patient in our study.

On multivariate logistic regression analysis, we found that older age, male sex, BMI, and preoperative TSH level were the only significant predictors of the need for hormonal therapy (Table 5).

**Table 5:** Multivariate logistic regression analysis to predict the need for hormonal therapy (n = 37). *: statistically significant at p-values <0.05. SE: standard error; CI: confidence interval; BMI: body mass index; HRT: hormonal replacement therapy; TSH: thyroid stimulating hormone

	Coefficient	SE	P	Odds ratio	95% CI
Age (years)	0.093	0.039	0.017*	1.10	1.0169-1.1845
Sex	1.705	1.859	0.359	5.50	0.1439-210.48
BMI (kg/m^2^)	1.925	0.745	0.010*	6.85	1.5924-29.484
Family history	2.757	2.306	0.232	15.760	0.171-1447.79
Duration of disease	0.351	0.360	0.330	1.4202	0.7013-2.8759
Clinical symptoms before HRT	3.876	2.674	0.147	48.26	0.255-9116.19
Preoperative TSH level (mIU/L)	3.234	1.470	0.028*	25.38	1.422-452.71

## Discussion

Restoring thyroid function is the aim of post-thyroidectomy replacement treatment, which aims to prevent over- or under-substitution by starting with the recommended dose of L-T4 seven days following the operation [14]. A dosage of L-T4 replacement that results in normal or slightly elevated serum T4 concentration and TSH concentration returning to a normal value is by definition the ideal dose [15,16].

Research suggests that an adult’s daily optimal dose of L-T4 should be 1.68 µg/kg. The adequacy of L-T4 was assessed using TSH concentration and the requirement for dose modification. For this reason, there is an unacceptably long lag between clinical symptoms and free T4 (FT4I) concentrations. It was shown that 74% of patients required HRT due to hypothyroidism [17].

Our study demonstrated that the patients who received an unadjusted dose of THR after thyroidectomy had abnormally low levels of TSH, with the mean preoperative TSH level of 1.9 ± 0.7 mIU/L. The mean postoperative TSH level after THR (unadjusted dose) was 0.29 ± 0.12 mIU/L. The patients involved in the second cycle who received an adjusted dose of HRT after intervention according to their weight had a normal preoperative TSH with a mean of 1.25 ± 0.11 mIU/L. After thyroidectomy and treatment with an adjusted dose of THR, they had a normal post-treatment TSH with a mean of 1.22 ± 0.12 mIU/L.

Sawka et al. [18] examined 40 patients receiving levothyroxine for primary hypothyroidism who also had depression symptoms. The patients were randomized by the investigators to receive T4 plus a placebo or T4/T3 combination therapy for 15 weeks. Their pre-study T4 dose was lowered by 50%, and 12.5 µg of T3 was added twice daily to accomplish combination therapy. Target TSH levels were maintained by adjusting the T4 and T3 dosages. Again, extensive evaluations of mood and level of well-being failed to find any variations in symptoms between the two therapy groups.

L-T4 is the treatment of choice for thyroidectomized patients, eliminates the symptoms of hypothyroidism in the majority of patients, and is highly effective at suppressing TSH in cases of thyroid cancer [9,19].

THR is not only essential for the restoration of endogenous thyroid hormones in cases of thyroid cancer but is also thought to inhibit tumor growth indirectly through its negative feedback mechanisms on pituitary TSH secretion subsequent to thyroidectomy.

On comparing 20 patients of the second cycle and 17 patients of the first cycle, the thyroid profile and calcium level after treatment in both groups of patients with an adjusted dose of THR revealed normal thyroid profiles and calcium levels with no significant difference between both groups.

The standard replacement therapy involves oral administration of T4 at a rate of 1.6 µg/kg/day, equating to 120 µg/day for an adult weighing 75 kg. However, daily doses vary from 50 to 200 µg, aiming to strike a balance between the risk of hyperthyroidism and the symptoms of hypothyroidism. The T4 dosage is influenced by the cause of hypothyroidism; for instance, moderate Hashimoto’s thyroiditis typically requires higher T4 doses than total thyroidectomy patients. Although approximately 80% of the ingested T4 is absorbed by the body, this percentage varies, affected by meal timing. Different components in generic T4 formulations may slightly alter absorption compared to brand-name versions. Despite established equivalences, it is advised that patients stick to a consistent brand throughout their treatment course [20].

A study by Peirce et al. [21] among patients who underwent total thyroidectomy and presented with severe hypothyroidism reported a gradual restoration of euthyroidism following sublingual administration of liquid L-T4. This finding establishes the formulation of liquid L-T4 as a viable alternative approach for the acute management of severe hypothyroidism. Kim et al. [22] revealed that levothyroxine medication was needed by 9.5% (95/192) of the participants to maintain euthyroidism.

Verhaert et al. identified a subset of patients for whom the initial postoperative dose of L-T4 was deemed adequate during their analysis. Six weeks after the procedure, a few adjustments to the first dose were found to be necessary for more than 50% of the patients who underwent partial thyroidectomy and were treated using a standard initial dose strategy that was not weight-related. This underscores the importance of endocrinological monitoring and the necessity to adjust the starting dosage [23].

Valenzuela et al. learned through a retrospective analysis of medical records of patients who underwent total thyroidectomy for benign disease that scheme A had a considerably diminished incidence of overdose, particularly among obese individuals. No statistically significant changes in the estimated THR dose were identified among the various schemes. The mean deviation in the calculated dosage across all schemes was 15 µg, which is equivalent to one dose. To prevent overdose, it may be essential to consider clinical criteria other than weight when determining the optimal dosage of THR [24].

On multivariate logistic regression analysis, we found that older age, male sex, BMI, and preoperative TSH level were the only significant predictors of the need for hormonal therapy.

Meyer et al. conducted a retrospective study involving 369 participants, revealing that 30% of these patients required replacement thyroid hormone. More than 39% initiated treatment beyond 12 months post-procedure, with 90% completing it within 36 months. Their findings underscored a strong correlation between age, preoperative TSH levels, and the need for THR. Age-related increases demonstrated a more than twofold likelihood of requiring replacement therapy, while a preoperative TSH level exceeding 2.5 mIU/L indicated an approximately fourfold heightened risk. Additionally, they observed a significant 4.5-fold probability of needing HRT compared to individuals with benign conditions, even after adjusting for preoperative TSH and age [13].

Previous studies have noted that individuals who developed early hypothyroidism had higher levels of TSH before surgery and a higher occurrence of thyroid peroxidase antibody positivity compared to those who experienced late-onset hypothyroidism. The preoperative TSH levels in euthyroid (normal thyroid level) patients were lower than those in patients who developed hypothyroidism after surgery [25].

Additional analysis of TSH levels in the Stoll et al. [26] study showed that when compared to patients with TSH levels ≤1.5 µIU/mL, patients with preoperative TSH levels between 1.51 and 2.5 µIU/mL had a significantly greater rate of hypothyroidism, while patients with TSH levels >2.5 µIU/L had an even higher rate. It was recently shown that following thyroid surgery, an increased TSH level constituted a separate risk factor for the development of hypothyroidism [27,28]. Furthermore, research employing a single threshold value, such as a TSH level greater than 1.6 or 2.0 µIU/mL, has revealed that individuals had a 7-10-fold increased risk of developing hypothyroidism after surgery [29,30].

Kim et al. [22] showed that rising preoperative TSH levels and female sex were independent predictors of the development of postoperative hypothyroidism, which was promptly addressed with hormone therapy.

One limitation of our study is its retrospective, single-institution design. Additionally, we noted variability in preoperative thyroid hormone level collection and postoperative TSH, which is common in many retrospective investigations.

## Conclusions

This clinical audit underscores a transformative shift in post-total thyroidectomy care, showcasing the efficacy of tailored HRT based on patient weight. Crucially, identifying key predictors influencing the need for therapy, i.e., age, sex, BMI, and preoperative TSH levels, offers a personalized treatment avenue. These findings hold promise for refining post-thyroidectomy care, emphasizing the need for personalized approaches in optimizing therapeutic outcomes and potentially reshaping standard care protocols. Further long-term studies are warranted to solidify these conclusions, but this study marks a pivotal step toward enhancing patient care and refining post-thyroidectomy management strategies.
